# Proteome changes in banana fruit peel tissue in response to ethylene and high-temperature treatments

**DOI:** 10.1038/hortres.2016.12

**Published:** 2016-04-27

**Authors:** Lina Du, Jun Song, Charles Forney, Leslie Campbell Palmer, Sherry Fillmore, ZhaoQi Zhang

**Affiliations:** 1 College of Horticulture, South China Agricultural University, GuangZhou, China; 2 Agriculture and Agri-Food Canada, Atlantic Food and Horticulture Research Centre, 32 Main St., Kentville, Nova Scotia B4N 1J5, Canada

## Abstract

Banana (*Musa* AAA group) is one of the most consumed fruits in the world due to its flavor and nutritional value. As a typical climacteric fruit, banana responds to ethylene treatment, which induces rapid changes of color, flavor (aroma and taste), sweetness and nutritional composition. It has also been reported that ripening bananas at temperatures above 24 °C inhibits chlorophyll breakdown and color formation but increases the rate of senescence. To gain fundamental knowledge about the effects of high temperature and ethylene on banana ripening, a quantitative proteomic study employing multiplex peptide stable isotope dimethyl labeling was conducted. In this study, green (immature) untreated banana fruit were subjected to treatment with 10 μL L^−1^ of ethylene for 24 h. After ethylene treatment, treated and untreated fruit were stored at 20 or 30 °C for 24 h. Fruit peel tissues were then sampled after 0 and 1 day of storage, and peel color and chlorophyll fluorescence were evaluated. Quantitative proteomic analysis was conducted on the fruit peels after 1 day of storage. In total, 413 common proteins were identified and quantified from two biological replicates. Among these proteins, 91 changed significantly in response to ethylene and high-temperature treatments. Cluster analysis on these 91 proteins identified 7 groups of changed proteins. Ethylene treatment and storage at 20 °C induced 40 proteins that are correlated with pathogen resistance, cell wall metabolism, ethylene biosynthesis, allergens and ribosomal proteins, and it repressed 36 proteins that are associated with fatty acid and lipid metabolism, redox–oxidative responses, and protein biosynthesis and modification. Ethylene treatment and storage at 30 °C induced 32 proteins, which were mainly similar to those in group 1 but also included 8 proteins in group 3 (identified as chitinase, cinnamyl alcohol dehydrogenase 1, cysteine synthase, villin-2, leucine-transfer RNA ligase, CP47 protein and calmodulin) and repressed 43 proteins in 4 groups (groups 4–7), of which 6 were associated with photosynthesis II oxygen-evolving protein, the photosynthesis I reaction center, sugar metabolism, the redox–oxidative system and fatty acid metabolism. Differences in the response to ethylene and holding temperature at 30 °C were also revealed and have been discussed. The identities and quantities of the proteins found were linked with quality changes. This study demonstrates that ethylene and high temperature influence banana fruit ripening and senescence at the proteomic level and reveals the mechanisms by which high temperature accelerates banana fruit ripening.

## Introduction

Banana (*Musa acuminate*, AAA group) is among the most popular fruits worldwide due to its attractive color, flavor, sweetness and texture. The banana is a typical climacteric fruit that produces a significant burst of autocatalytic ethylene and respiration at the onset of ripening, followed by changes in color, firmness and flavor.^1^ Banana fruit are typically harvested at the green mature stage and shipped to wholesale markets, where they are then treated with ethylene to stimulate fruit ripening; this treatment results in a rapid color change from green to yellow and the development of a ‘fruity’ flavor when stored at 16–24 °C.^2^ However, the normal de-greening process can be interrupted if the fruit are stored at temperatures above 24 °C. Under this condition, fruit soften normally but remain green, yielding ‘green ripe’ bananas.^3–5^ This response of banana fruit to high post-harvest temperatures, which results in the failure of proper peel color to develop, causes significant economic loss for the banana industry.

Chlorophyll degradation commonly occurs during leaf senescence and fruit ripening in plants. Since the 1990s, research in understanding chlorophyll degradation in leaves has advanced greatly.^6–8^ Chlorophyll degradation involves a group of chloroplast enzymes including chlorophyll *b* reductase, 7-hydroxymethyl chlorophyll *a* reductase and magnesium dechelatase.^9–11^ During this degradation, chlorophyll *a* loses Mg and forms pheophorbide *a,* which is further catabolized by pheophorbide *a* oxygenase to form red reducing fluorescence compounds termed fluorescent chlorophyll catabolites (FCCs). Pheophorbide *a* oxygenase further oxidizes FCCs to red chlorophyll catabolites. Primary FCCs are exported from the chloroplast to the cytosol where they can be modified enzymatically. Most FCCs are imported into the vacuole where they are isomerized to colorless compounds.^8^ Research indicates that chlorophyll breakdown in ripening fruit, such as apple and pear, is similar to that in senescent leaves. However, banana fruit present a different chlorophyll degradation pathway, in which large amounts of FCCs with blur luminescence accumulate in the outer region of the peel.^12^ This finding has been recognized as a critical step in chlorophyll degradation.^8^ ‘Green ripe’ bananas, which occur when bananas are held at temperatures >24 °C, is recognized as a physiological disorder by the banana industry.^2,3^ However, in another family of banana (*Musa*, ABB group), normal chlorophyll degradation occurs at 35 °C.^13^ Other fruit, such as tomato^14^ and ‘Shamouti’ orange,^15^ also demonstrate normal chlorophyll degradation at 35 °C. Therefore, the mechanism by which temperature alters the degradation of chlorophyll in banana is unknown. Banana fruit also express rapid senescence and high ethylene production when held at high temperatures; however, exogenous ethylene treatment does not accelerate chlorophyll degradation.^2^ A comparative study on banana fruit that were stored at 20 or 30 °C revealed that the conversion of starch to sugar is significantly enhanced at 30 °C.^16^ Similarly, the rapid accumulation of soluble solids has also been associated with ‘green ripe’ bananas.^4^ When the gene expression associated with chlorophyll degradation in bananas held at 20 and 30 °C was compared, the senescence of fruit held at 30 °C was accelerated but the expression of genes related to chlorophyll degradation was inhibited.^4^

As a typical climacteric fruit, banana shows not only a climacteric change in ethylene production and respiration but also a dose response to ethylene treatment; for this reason, banana has been recognized as a model fruit for post-harvest physiology studies.^17,18^ Significant research has been reported at both the metabolomic and genomic levels that show the effects of fruit ripening and ethylene treatment on fruit quality indices of banana fruit, including, color,^19^ firmness, cell wall composition^20^ and aroma/flavor production.^21,22^ Ripening-related gene expression in banana peel was examined and compared with that from fruits that were ripened at 20 and 35 °C. A putative ATP-citrate lyase clone was isolated from ripening peel and was downregulated at 35 °C.^19^

At the proteomic level, 50 proteins using two-dimensional gels that respond to fruit ripening and ethylene signal transduction have been identified.^23,24^ Another study identified class I chitinase, lipid transfer protein, thaumatin-like protein and 1,3-glucanase as a group of allergens in banana.^25^ However, more comprehensive studies employing quantitative proteomic techniques on banana fruit have not been reported. In this study, a quantitative proteomic method employing multiplex peptide stable isotope dimethyl labeling was applied to reveal changes in protein levels in response to ethylene and storage temperature to further investigate the mechanism of the ‘green ripe’ phenomena at the proteomic level.

## Materials and methods

### Fruit samples and treatments

Mature green banana fruit (*Musa* spp. AAA group, Cavendish subgroup) that had not been treated with ethylene were purchased from a local wholesale market. For each biological replicate, 20 kg of banana fruit (7–9 hands) were obtained. From each hand, five fruit were randomly selected. The fruit samples were prepared and treated as described previously.^26^ Briefly, the fruit were divided into four treatments. Two groups were placed in 15-L glass chambers and exposed to a 0.42 mL s^−1^ flow of 10 μL L^−1^ ethylene for 24 h at 20 °C to initiate ripening. The other two groups were placed in a chamber with air and no ethylene at 20 °C for 24 h. After each treatment, one group of fruit from each treatment was allowed to ripen at 20 °C in unsealed polyethylene plastic bags for 1 day, and the remaining two groups were ripened similarly at 30 °C. Five individual fruit were picked from each group, and fruit color and chlorophyll fluorescence (CF) were measured at three different locations on the peel from bottom to top. CF was measured using an OS-500 modulated Fluorometer (OPTI Sciences, Hudson, NH, USA) operating in the ‘Fv/Fm’ mode. Fo (minimum fluorescence), Fv (variable fluorescence), Fm (maximum fluorescence) and Fv/Fm (exciton transfer efficiency) were determined after dark-adaptation for 30 min at room temperature.^26^ Peel tissues were then sampled, separately sliced, mixed and frozen using liquid N_2_. The frozen tissue was then pulverized and stored at −85 °C until further use.

### Protein extraction, quantitation and digestion

Proteins were extracted using a modified phenol extraction followed by ammonium acetate–methanol precipitation.^27^ Proteins were quantitated using the RC/DC protein assay kit (Bio-Rad Laboratories, Hercules, CA, USA); bovine serum albumin was used as the standard.^28^ Detailed procedures for protein digestion and peptide desalting have been described.^29,30^ Briefly, the protein samples were diluted with triethylammonium bicarbonate to a final concentration of less than 1 m urea, reduced with dithiothreitol for 30 min at 30 °C and then alkylated with iodoacetamide for 60 min at room temperature in the dark. The proteins were digested with sequencing grade-modified trypsin (Promega, Madison, WI, USA) overnight at 37 °C (final trypsin to substrate ratio of 1:50 (w/w). The peptides were desalted using Oasis HLB cartridges (Waters, Milford, MA, USA). The eluted peptides were then evaporated to dryness using a vacuum centrifuge and re-dissolved in 2% (v/v) ACN and 1% (v/v) formic acid.

### Protein identification and quantitation using stable isotope dimethyl labeling

The resulting peptides of the total tryptic digestion of the various biological samples were labeled with isotopomeric dimethyl labels as described by Boersema *et al.*^31^ with minor modifications.^32^ Due to the labeling regime, only three treatment samples could be compared. Bananas that had not been treated with ethylene but had been stored for 2 days at 20 °C were used as the control, while the test samples were ethylene-treated bananas that had been stored for 1 day at 20 °C and 30 °C, respectively. Four hundred (400) μg of protein obtained from samples of each treatment group were re-suspended in 200 μL of IPG buffer comprising 5% glycerol and ampholytes at pH 3.0–10.0 (GE Healthcare, Bio-Sciences AB, Rapsgatan 7E, Uppsala, Sweden). Samples of fruit, including control fruit without ethylene treatment at 20 °C for 2 days, were labeled as ‘light’ (L), samples obtained after 1 day of ethylene treatment at 20 °C were labeled as ‘intermediate’ (M) and samples obtained after 1 day of ethylene treatment at 30 °C were labeled as heavy’ (H), following incubation with CH_2_O (light formaldehyde), CD_2_O (intermediate formaldehyde) and ^13^CD_2_O (heavy formaldehyde), respectively. Two labeled groups were created from two biological replicates. After labeling, three differentially labeled samples were pooled at a ratio of 1:1:1 (w/w) and desalted prior to OFFGEL peptide fractionation (Supplementary Figure S1).

### OFFGEL fractionation

Total labeled peptides were fractionated into 24 fractions based on *pI* using an OFFGEL-Fractionator (3100, Agilent Technologies, Palo Alto, CA, USA) according to the manufacturer’s protocol and as described previously.^30^

### LC/MS analysis; Q-TOF

Details of the LC/MS procedure using q-TOF have been described previously.^32^ Briefly, mass spectra were acquired on a Waters Quadrupole Time-of-flight (QTof) liquid chromatography/mass spectrometry (LC/MS) instrument (XeVo, Milford, MA, USA), which was equipped with a nano-electrospray source and a nano-lockspray interface. A lock mass solution of [Glu^1^]-Fibrinopeptide B (200 fmol μL^−1^) was infused at a frequency of 60:1 (sample-to-reference ratio). A potential of 2800 V was applied to a 20-μm-inner diametre, tapered electrospray tip (FS360-75-30-N-C12, New Objective, Woburn, MA, USA). The cone voltage was set to 30 kV, and the source temperature used was 100 °C. Chromatographic separations were conducted using a nano-Acquity UPLC system (Waters) equipped with an UPLC peptide BEH C_18_ column (75 μm×10 cm×1.7 μm; Waters) in a data-dependent mode. Separation was carried out using a gradient of 5% B to 30% B over 53 min, then increasing to 90% B over 5 min at a flow rate of 400 nL min^−1^, where A: 0.1% formic acid in water and B: 0.1% formic acid in acetonitrile.

Raw data files from the 24 OFFGEL fractions for each labeling group were combined, and a single database search was performed. Peptide sequence searches were conducted using MASCOT version 2.5.0 (Matrix Science, London, UK) to search the NCBI using the following parameters: raw MS/MS data were searched against NCBI *viridiplantae* entries, 2 474 089 sequences, updated on 01 December 2014 (NIH, Bethesda, MD, USA). This updated sequence database also contains 43 900 protein sequences for banana. The MS and MS/MS mass tolerances were both 0.3 Da, and one missed cleavage was allowed. Carboxamidomethyl cysteine and oxidized methionine were set as fixed and variable modifications, respectively. Proteins with significant peptide matches were selected for error tolerant searching. MASCOT searches were based on a significance threshold of *P<*0.05, and the MudPIT scoring system (MASCOT scores ⩾52) was used. False positive peptide identification rates were calculated using the decoy option provided by MASCOT and were estimated as 1.0%. Automated quantification was performed using MASCOT Distiller version 2.5.1.0 (Matrix Science) to calculate the mean protein ratio and s.d. The raw data were also manually inspected.

### Statistical analysis

Four biological experiments were conducted on quality and postharvest physiology. For the proteomic study, two biological replicates were employed. To determine protein function, the proteomic GO (Gene Ontology) tool was used (http://blast.ncbi.nlm.nih.gov/). Protein clustering and heatmap profiles were produced using EPCLUST software (http://www.bioinf.ebc.ee/EP/EP/EPCLUST/) and ClustVis (http://biit.cs.ut.ee/clustvis/). Protein sequences were also searched against the NCBI database employing BLASTp and cut off values of *E*-value 1×10^−5^. To characterize the GO function group, an enriched analysis was conducted using common identified proteins as a reference.

## Results and Discussion

### Physiological changes after ethylene and high-temperature ripening

Physiological changes in banana fruit in response to ethylene treatment and storage at 20 and 30 °C were evaluated over 7 days as reported in a previous study.^26^ The ‘green ripe’ phenomenon was observed 4 days after ethylene treatment and high-temperature storage at 30 °C; the hue angle remained >105° compared with <96° for fruit stored at 20 °C. These differences were not apparent after 1 day of storage at 20 and 30 °C, where hue angle was decreased from 119° to 115° and 114°, respectively. Fruit without ethylene treatment remained green. Ethylene and high-temperature ripening caused significant changes in chloroplast function as measured by peel CF.^26^ Among the CF parameters, Fv/Fm is an indicator of photochemical efficiency. Without ethylene treatment, no change in Fv/Fm was observed (the value remained at ~0.81). With ethylene treatment, however, Fv/Fm decreased to 0.67 and 0.53 following 1 day of storage at 20 °C and 30 °C, respectively, indicating a loss of photochemical efficiency. Fo increased from 296 to 385 at 30 °C, which has been proposed to reflect the effect of stress on chloroplast function.^33^

### Protein identification and cluster analysis of proteins that exhibited altered expression levels

Significant changes in protein levels in response to ethylene and 30 °C treatments were revealed through a quantitative proteomic investigation that employed peptide dimethylation; this study simultaneously quantified the proteome of three samples with different biological changes that were subjected to both ethylene and storage at 30 °C. A total of 1067 and 1028 proteins were identified and quantified from two individual biological replicates. Among these proteins, 416 were common to both replicates, and of these, the expression of 91 were changed twofold or more in response to treatment; this value (twofold) was used as a cutoff value to distinguish significant increases and decreases of protein abundance when compared with the control.

**Figure 1:**
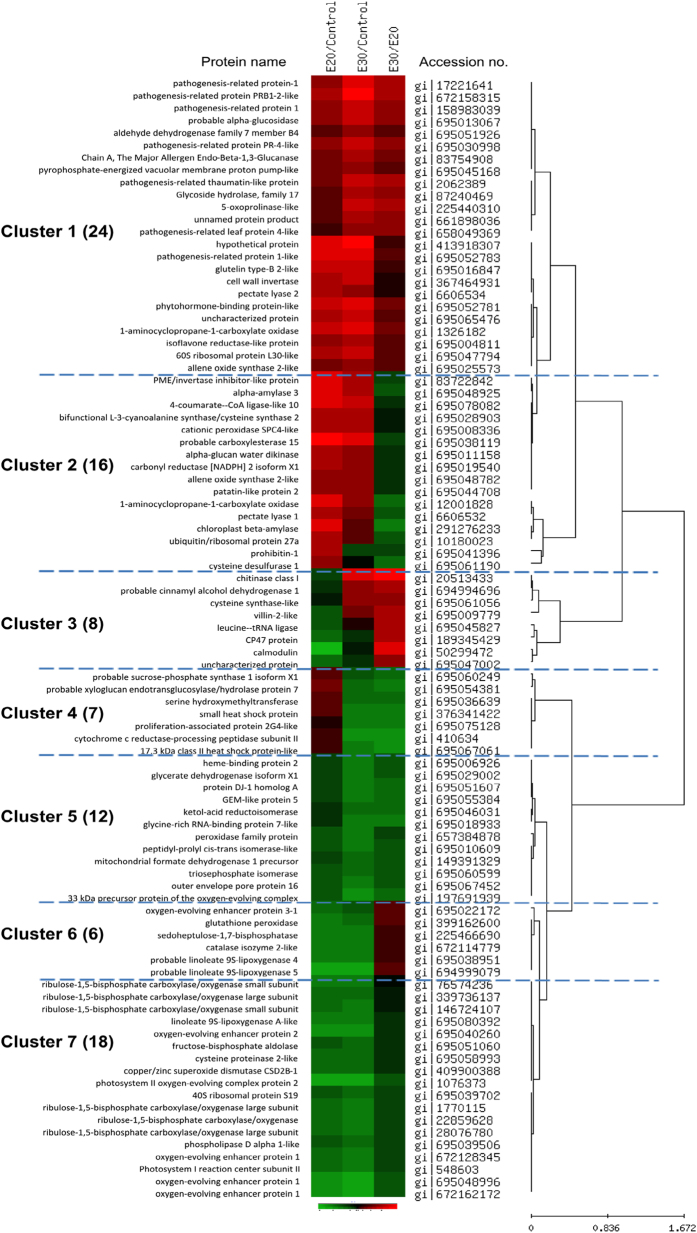
Hierarchical cluster analysis of 91 protein abundances in banana fruit peel tissue after ethylene treatment and stored for 1 day at 20 °C or 30 °C. On the basis of the abundance ratio between ethylene-treated fruit at 20 °C versus control, ethylene-treated fruit at 30 °C versus control as well as ethylene-treated fruit at 30 °C versus ethylene-treated fruit at 20 °C, seven major protein clusters were demonstrated. Cluster 1 includes proteins that were increased in abundance at both 20 °C and 30 °C; Cluster 2 includes proteins that were increased in abundance at both 20 °C and 30 °C, but to a lesser degree at 30 °C; Cluster 3 includes proteins that were decreased in abundance at 20 °C but were present at higher abundance in 30 °C; Cluster 4 includes proteins that were increased in abundance at 20 °C but were present at lower abundance at 30 °C; Cluster 5 includes proteins that were decreased in abundance at both 20 °C and 30 °C and exhibited lower abundance at 30 °C; Cluster 6 includes proteins that were decreased in abundance at both 20 °C and 30 °C and presented at higher abundance at 30 °C; and Cluster 7 includes proteins that were decreased in abundance at both 20 °C and 30 °C but were of similar abundance at both of these temperatures. The protein name is indicated on the right side of the plot and is listed in Table 1. Increasing intensities of red or green color indicate differential up- or down-regulation compared with the control at day 0, respectively. Identified proteins are also marked and listed in Table 1.

Seven significant clusters were identified and categorized for these 91 proteins (Figure 1 and Table 1). Cluster 1 comprised 24 proteins that included: PR-1, PR-4-like, pathogenesis-related protein PRB1-2, pathogenesis-related protein 1, alpha-glucosidase Os06g0675700, aldehyde dehydrogenase family 7 member B4, major allergen, endo-beta-1,3-glucanase, pyrophosphate-energized vacuolar membrane proton pump-like, cell wall invertase, pectate lyase 2, phytohormone-binding protein-like, 1-aminocyclopropane-1-carboxylate oxidase, isoflavone reductase, 60S ribosomal protein L30 and allene oxide synthase 2-like proteins, all of which increased after ethylene treatment and storage at 20 or 30 °C for 1 day. The increase in these proteins was greater at 30 °C than at 20 °C. Cluster 2 comprised 16 proteins that increased after ethylene treatment and 1 day of storage at 20 and 30 °C, but the increase was greater at 20 °C than at 30 °C. Proteins in Cluster 2 included: pectin methyl esterase/invertase inhibitor-like protein; alpha-amylase 3; 4-coumarate-CoA ligase-like 10; bifunctional l-3-cyanoalanine synthase/cysteine synthase 2, mitochondrial; cationic peroxidase SPC4-like; carboxylesterase 15; alpha-glucan water dikinase; allene oxide synthase 2-like; patatin-like protein 2; another ACO; pectate lyase 1; beta-amylase; ubiquitin/ribosomal protein 27a; prohibitin-1; and cysteine desulfurase 1. Cluster 3 included eight proteins, the levels of which decreased following ethylene treatment and 1 day of storage at 20 °C but increased when stored at 30 °C compared with those stored at 20 °C. Proteins in Cluster 3 included: chitinase class I; cinnamyl alcohol dehydrogenase 1; cysteine synthase; villin-2; leucine-transfer RNA ligase; CP47 protein and CaM. Cluster 4 comprised seven proteins, including sucrose-phosphate synthase 1, xyloglucan endotransglucosylase/hydrolase protein 7, serine hydroxymethyltransferase, small HSP, proliferation-associated protein 2G4-like, cytochrome c reductase-processing peptidase subunit II and 17.3 kDa class II HSP. This group of proteins increased following ethylene treatment and 1 day of storage at 20 °C but decreased when stored for 1 day at 30 °C. Clusters 5, 6 and 7 included 36 proteins, the levels of which decreased following ethylene treatment regardless of storage temperature. Proteins in Cluster 6 were slightly more abundant after storage at 30 °C than at 20 °C. These significant changes in protein abundance revealed dynamic changes in protein profiles in response to ethylene and storage temperature.

### Protein changes in relation to metabolic pathways

A previous study that employed a differential gene expression analysis indicated 20 non-redundant families of clones that were significantly changed in banana fruit in response to ethylene treatment; these genes included those encoding enzymes involved in ethylene biosynthesis, respiration, starch metabolism, and cell wall degradation.^19,34^ Elevated temperature at 35 °C for 2 and 6 days had little or no effect on the expression of most cDNA clones isolated from banana peel, indicating that the effect of high temperature on chlorophyll catabolism is unlikely to be mediated through a general inhibition of ripening.^19^ However, ATP-citrate lyase was found to be down-regulated, and it was postulated that new fatty acid biosynthesis occurs during the conversion of chloroplasts to chromoplasts and ATP-CL might play a role in such events. In this quantitative protein investigation of 413 proteins, 91 significantly changed and were identified and quantified. Proteins were categorized as belonging to 9 biological processes according to the GO annotation (Supplementary Figure S2), representing various biological functions including chloroplast and photosynthesis function, redox and reactive oxygen species (ROS) scavenging, stress response, and cell wall, signal, fatty acid, amino acid, starch and sugar metabolism. These proteins with significantly changed abundance might be related to fruit metabolism and could lead to a better understanding of the physiological changes involved in ‘green ripe’ and fruit ripening of banana fruit.

#### Chloroplast and photosynthesis

**Figure 2:**
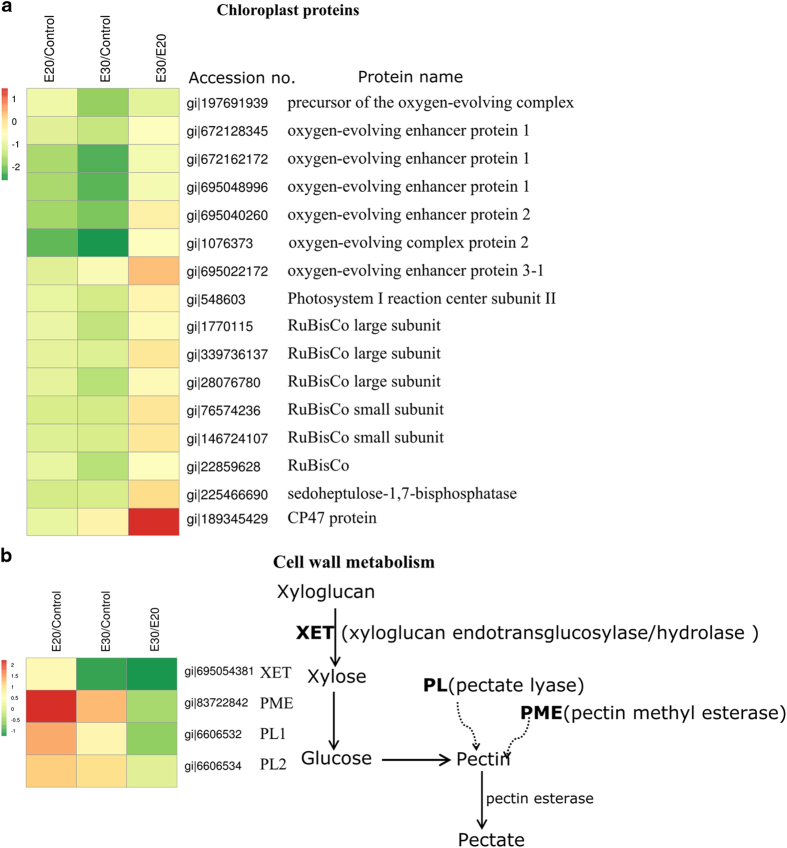
Proteins with significantly changed abundances as classified into putative functional group category—(**a**) chloroplast proteins; (**b**) cell wall metabolism.

The proteins that were present at significantly changed abundance and were involved with chloroplast and photosynthesis function included oxygen-evolving enhancing proteins, proteins associated with the photosystem I reaction center subunit, Rubisco, and CP47. The abundances of most of these proteins were either unchanged or decreased in response to ethylene and storage at 20 and 30 °C (Figure 2a). Only CP47 was increased in abundance in response to storage at 30 °C compared with 20 °C. CP47 is one of the core antenna proteins in the photosynthesis II system and interacts with the oxygen-evolving site of photosynthesis II and manganese-stabilizing protein.^35^ However, most of the proteins that were related to oxygen-evolving enhancing proteins were inhibited by both ethylene and storage at 30 °C. The development of oxygenic photosynthesis brought about many changes that required alterations to existing pigments, the generation of an oxygen evolution complex, and protection against the toxic effects of oxygen by-products. Therefore, a decrease of oxygen-evolving enhancing proteins indicates reduced photosynthetic activity, which might be linked to the reduced photosynthetic capacity observed here by the decrease in CF-related parameters (Fv/Fm) and the increase in Fo.

A previous biochemical analysis reported that when banana peel ripened normally at 20 and 35 °C, chlorophyll breakdown products could be isolated from fruit. The colored breakdown products, chlorophyllide and pheophorbide, were not detected in peel that had been subjected to either of the two temperature treatments.^19^ However, non-fluorescence chlorophyll catabolites accumulated to higher concentrations at 20 °C than at 35 °C.

#### Cell wall metabolism

A group of proteins involved in cell wall metabolism including xyloglucan endotransglucosylase,^36^ two pectin lyases and pectin methyl esterase were found to increase in ethylene-treated banana after storage at 20 °C. A lesser increase was found in fruit peel that had been stored at 30 °C (Figure 2b). The increase in the abundance of these proteins might explain the significant loss of firmness of ‘green ripe’ banana fruit after ethylene treatment, a common phenomenon in bananas.^4,26^ For cassava root during early program cell deterioration, it was reported that the endotransglucosylase hydrolase (XET) and pectin methyl esterase were upregulated, which indicated a remodeling of cell wall components.^37^ This result is consistent with similar findings for ethylene-treated banana fruit and might be common at senescence.

#### ROS scavenging and stress response

**Figure 3:**
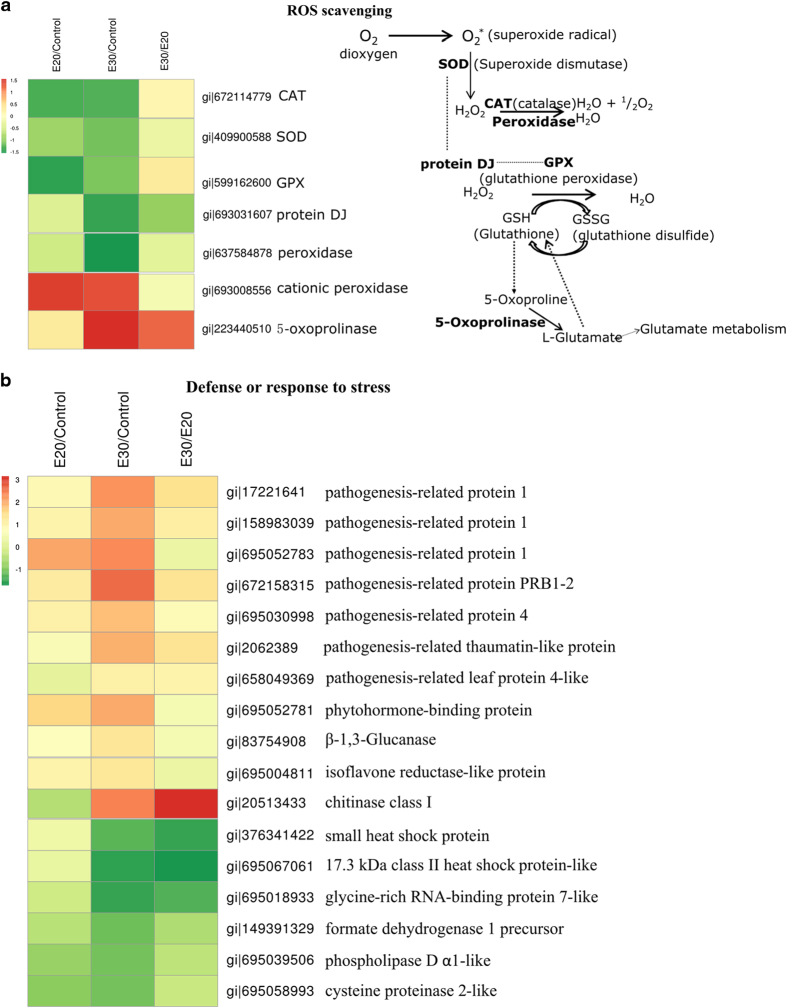
Proteins with significantly changed abundances as classified into putative functional group category—(**a**) ROS scavenging; (**b**) defense or response to stress.

With ROS scavenging proteins, a significant decrease of catalase (CAT), superoxide dismutase and glutathione peroxidase was observed in ethylene-treated fruit held at both 20 and 30 °C (Figure 3a). In contrast, the abundance of cationic peroxidase was increased at both temperatures, whereas 5-oxoprolinase was more abundant in fruit held at 30 °C (Figure 3a). The decreases in the abundances of CAT, superoxide dismutase and glutathione peroxidase indicated a possible down-regulation of ROS scavenging capacity under these treatments. Proteomic studies in apples showed that superoxide dismutase and glutathione peroxidase increased with fruit ripening and in response to ethylene treatment, but CAT tended to decrease.^29^ In cassava root, both superoxide dismutase and CAT increased during post-harvest physiological deterioration. The increases in the abundances of cationic peroxidase and 5-oxoprolinase in fruit held at 30 °C indicated that ROS scavenging and glutathione (GSH) glutamate metabolism are linked. In Arabidopsis, the role of 5-oxoprolinase in GSH degradation to glutamate has been reported.^38^ One of the roles of GSH degradation is to provide Cys for protein synthesis and as a precursor for numerous metabolites; this might explain why cysteine synthase was increased in conjunction with ethylene and storage at 30 °C. The result obtained implies that GSH degradation in banana peel forms part of the response to ethylene and 30 °C storage.

A group of stress response proteins was also found to increase in abundance after treatment with ethylene and storage at 30 °C (Figure 3b). These proteins include various pathogenesis-related proteins, phytohormone-binding protein, β-1,3-glucanase, isoflavone reductase and chitinase class I protein. Significant increases in the abundances of pathogenesis-related protein, β-1,3-glucanase and chitinase I proteins have been reported in gene expression^34^ and proteomic studies,^24^ indicating that these proteins respond to both fruit ripening and 30 °C storage. In contrast, the abundances of heat shock proteins (HSP) and glycine-rich RNA-binding protein decreased in response to ethylene and holding at 30 °C. Formate dehydrogenase, phospholipidase and cysteine proteinase 2 decreased in response to ethylene treatment, but showed only little change with holding at 30 °C compared with 20 °C, demonstrating a possible downregulation of these proteins after ethylene treatment.

#### Signaling pathway

**Figure 4:**
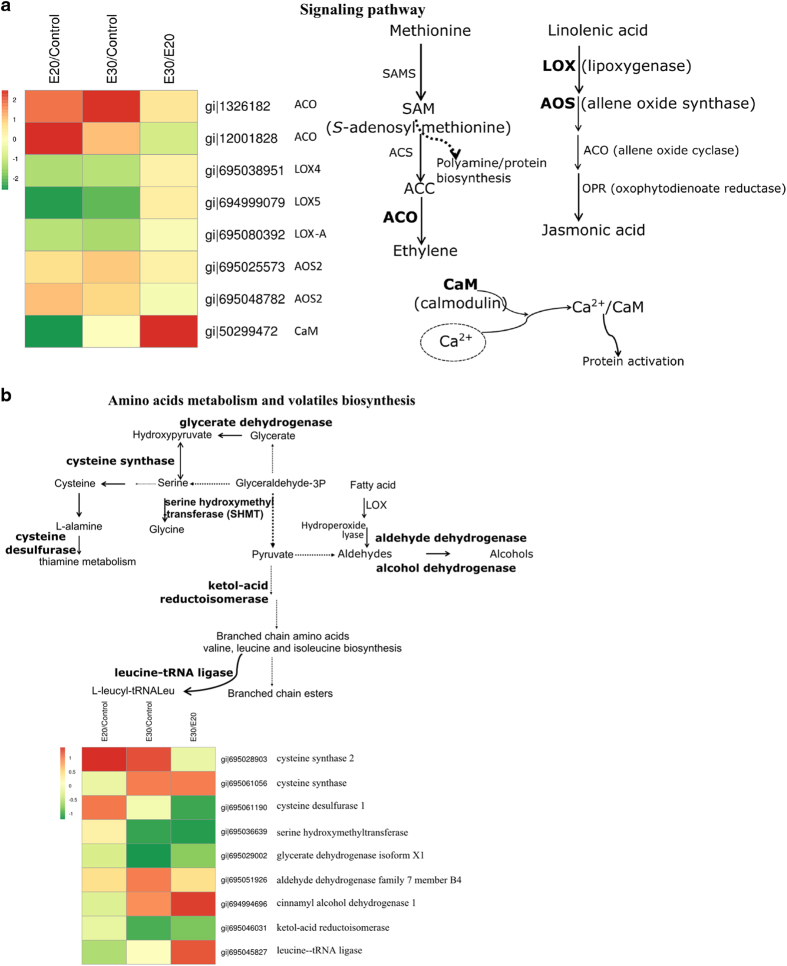
Proteins with significantly changed abundances as classified into putative functional group category—(**a**) signal pathway; (**b**) amino acid metabolism and volatile compound biosynthesis.

In terms of signaling pathways, two ACOs and AOS increased in response to ethylene treatment and storage at 30 °C (Figure 4a), whereas three LOX proteins (LOX4, LOX5 and LOXA) and CaM decreased. LOX proteins have been found to be important in fruit ripening and flavor formation in apples^39,40^ and in other fruit, including tomato^41^ and peach.^42^ In banana fruit, differential expression of LOX is observed between peel and pulp tissue after ethylene treatment, with an overall decrease in abundance in the peel.^26^ The proteomic data obtained here confirm the previously reported *LOX* gene expression data. CaM has an important role in plant stress responses, and its gene expression is often induced by various stresses.^43^ CaM expression has an important signal transduction role during heat stress, and several HSPs are expressed in correlation with the accumulation of CaM transcripts and proteins in plants.^44^ However, we found that one small HSP and one 17.3 kDa class II HSP decreased in banana peel after ethylene treatment and during ripening at 30 °C (Figure 3b). It has been reported that seven genes encoding calcium/CaM-regulated proteins respond differently to various stress signals (cold, wounding, pathogen infection, salicylic acid and methyl jasmonate treatment) in mature green tomato fruit. Among these genes, only one exhibited increased expression in response to all treatments, a finding that indicated that individual genes in this Solanum lycopersicum stress responsive (SlSRs) has a distinct role in response to stress and might act as a coordinator between calcium and other stress signals.^45^ Little is known about CaM in banana fruit, and the decrease of CaM abundance in response to ethylene warrants further investigation.

#### Amino acid metabolism and volatile biosynthesis

Proteins involved with amino acid metabolism were also found to increase in response to ethylene treatment and storage at 30 °C. These proteins included cysteine synthase, cysteine desulfurase, serine hydroxymethyltransferase, glycerate dehydrogenase isomer, keto-acid reductoisomerase and leucine–transfer RNA ligase (Figure 4b). Among these proteins, one of the cysteine synthases 2 increased in response to both ethylene and storage at 30 °C; another cysteine synthase increased in abundance only in response to holding at 30 °C. Cysteine desulfurase increased in response to ethylene but not in response to storage at 30 °C. Cysteine and related compounds not only have important roles in plant metabolism (including the synthesis of defense compounds) but also act as signals for regulating essential plant processes, such as the redox signaling process, photosynthesis, plant protection, plant immunity and root development; this signaling occurs in various cellular compartments.^46,47^

Cysteine desulfurase is a pyridoxal 5’-phosphate-dependent enzyme that catalyzes the conversion of l-cysteine to l-alanine and sulfane sulfur. This group of proteins participates in various biological processes including electron transfer, the regulation of gene expression, iron–sulfur storage photosynthesis and other enzyme activities.^48,49^ The identified proteins Cys synthase 2 and Cys desulfurase are located in the mitochondria. Cys synthase 2 acts as a l-3-cyanoalanine synthase, which catalyses the production of cyanide in response to ACO increase as a form of detoxification. Another cysteine synthase protein responded to high temperature but not to ethylene. This result confirms the association between increased levels of 5-oxoprolinase (responsible for GSH degradation) with glutamate, resulting in a possible increase of Cys as mentioned in the previous section describing ROS. This indicates that a complex system is used to homeostatically maintain Cys for normal plant function and in response to both biotic and abiotic stresses. The increases in Cys synthase and Cys desulfurase abundances in response to ethylene treatment might indicate the need to balance Cys levels in banana peel and to compensate for the Cys increase that occurs in response to storage at 30 °C. Our proteomic study on changes in the abundance of Cys-related proteins in response to ethylene treatment and storage at 30 °C sheds light on the importance of these groups of proteins in plant metabolism. However, further investigation on cysteine metabolism during banana fruit ripening and in response to various stresses is warranted.

Previous studies revealed changes in the gene expressions of alcohol acyl transferase, aldehyde dehydrogenase, pyruvate dehydrogenase, hydroperoxide lyase (HPL), lipoxygenase^50^ and branched-chain amino acid aminotransferase (BCAT), which have important roles in the formation of volatiles;^26^ these enzymes were increased during fruit ripening, especially in response to ethylene treatment. Our proteome study found significant increases in aldehyde dehydrogenase family 7 proteins, which catalyze the conversion between aldehydes and alcohols.

#### Starch and sugar metabolism

**Figure 5:**
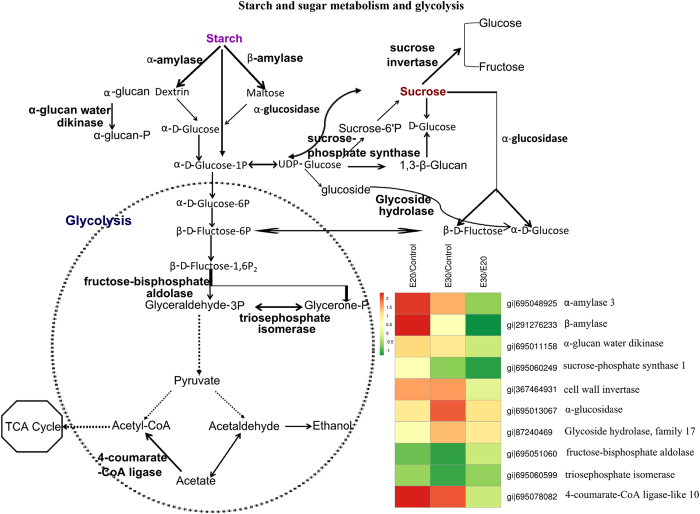
Proteins with significantly changed abundances as classified into putative functional group category—starch and sugar metabolism, as well as glycolysis.

Significant changes in proteins involved with starch and sugar metabolism were also found. α- and β-amylases as well as glucan water dikinase increased in banana fruit that were treated with ethylene and held at 30 °C (Figure 5). In contrast, fructose-bisphosphate aldolase and triosephosphate isomerase were decreased in abundance. At 30 °C, however, the abundances of most of these proteins were decreased to a greater extent than they were decreased at 20 °C.

In addition, the abundance of 16 proteins, glutelin type-B 2-like, paratin-like protein 2, prohibitin-1, mitochondrial-like isoform X1, phytohormone-binding protein, and villin-2-like proteins were increased after ethylene treatment and were further increased when held at 30 °C. The abundances of cytochrome c reductase-processing peptidase subunit II, MPP subunit II, P53 protein, outer envelope pore protein 16, 40S ribosomal protein S19, and proliferation-associated protein 2G4 were changed slightly in response to ethylene but were decreased significantly when held at 30 °C compared with 20 °C, indicating these proteins are sensitive to high-temperature storage.

## Conclusions

Banana fruit peel tissue showed significant physiological changes in color and CF after ethylene treatment and storage at 20 and 30 °C. Decreases of Fv/Fm were observed, indicating decreased photosynthetic capacity in peel tissue. Peptide dimethylation in combination with the OFFGEL technique is an effective and high throughput approach that can identify and quantify a large number of proteins and reveal their response to ethylene and storage temperature in banana peel tissue. Significant changes in proteins abundance contribute to the abnormal ripening of banana peel under these conditions. Among the proteins exhibiting significantly changed abundance, multi-pathway proteins such as those involved in chloroplast function, ROS scavenging, signal pathways, defense and the stress response, as well as proteins involved in cell wall, amino acid and sugar metabolism were affected by the treatments. These results revealed at the protein level the molecular metabolism that contributes to abnormal peel color when fruit are held at 30 °C. The identified proteins can be used as targets for further proteomic analysis using MRM to investigate their role, both individually and as a group, in the response of banana fruit to ethylene treatment and high temperature.

## Figures and Tables

**Table 1:** Significantly changed protein abundances in banana peel in response to ethylene treatment and ripening for 1 day at 20 and 30 °C

*No.*	*Accession*	*Description*	*Mass (Da)*	*Score*	*Peptide count*	*E20/control*	*S.D.*	*E30/control*	*S.D.*	*E30/E20*	*S.D.*
*Cluster 1 (24)*											
1	gi|17221641	PR-1 (*Solanum torvum*)	19 196.45	177	10	1.88	0.37	4.79	1.49	2.68	1.32
2	gi|672158315	Pathogenesis-related protein PRB1-2-like (*Phoenix dactylifera*)	17 391.8	182	8	2.25	0.45	6.05	3.53	2.58	1.05
3	gi|158983039	Pathogenesis-related protein 1 (*Musa acuminata*)	17 696.82	1086	27	2.00	1.02	4.05	0.99	2.18	0.62
4	gi|695013067	Probable alpha-glucosidase (*Musa acuminata* subsp. malaccensis)	99 173.86	207	8	1.93	0.58	3.84	0.32	2.05	0.45
5	gi|695051926	Aldehyde dehydrogenase family 7 member B4 (*Musa acuminata* subsp. malaccensis)	55 197.62	134	4	1.41	0.24	2.01	0.46	1.42	0.08
6	gi|695030998	Pathogenesis-related protein PR-4-like (*Musa acuminata* subsp. malaccensis)	16 044.13	941	23	2.08	0.26	3.58	1.00	1.76	0.70
7	gi|83754908	β-1,3-Glucanase (*Musa acuminata*)	33 460.41	6634	153	1.70	0.03	2.55	0.55	1.49	0.30
8	gi|695045168	Pyrophosphate-energized vacuolar membrane proton pump-like (*Musa acuminata* subsp. malaccensis)	80 723.42	603	17	1.44	0.76	2.07	1.65	1.32	0.45
9	gi|2062389	Pathogenesis-related thaumatin-like protein (Oryza sativa)	25 675.46	540	11	1.50	0.04	3.87	1.19	2.59	0.87
10	gi|87240469	Glycoside hydrolase, family 17 (Medicago truncatula)	40 402.13	90	5	1.31	0.08	2.55	1.37	1.92	0.93
11	gi|225440310	5-Oxoprolinase-like (Vitis vinifera)	139 321.6	104	4	1.28	0.05	2.95	1.97	2.34	1.63
12	gi|661898036	Unnamed protein product (Coffea canephora)	25 049.74	177	6	1.26	0.13	2.47	1.02	2.01	1.01
13	gi|658049369	Pathogenesis-related leaf protein 4-like (Malus domestica)	20 406.33	170	10	1.18	1.17	2.12	1.91	1.96	0.33
14	gi|413918307	Hypothetical protein (Zea mays)	26 204.45	108	2	5.26	4.54	6.45	6.16	1.15	0.18
15	gi|695052783	Pathogenesis-related protein 1-like (*Musa acuminata* subsp. malaccensis)	17 679.15	593	32	4.17	0.15	5.11	0.85	1.22	0.16
16	gi|695016847	Glutelin type-B 2-like (*Musa acuminata* subsp. malaccensis)	38 469.36	520	43	3.11	0.05	3.67	1.55	1.18	0.48
17	gi|367464931	Cell wall invertase (*Musa acuminata* AAA Group)	66 197.93	139	10	2.86	0.65	2.95	0.31	1.05	0.13
18	gi|6606534	Pectate lyase 2 (*Musa acuminata* AAA Group)	49 927.86	476	20	2.27	0.73	2.05	1.35	1.05	0.93
19	gi|695052781	Phytohormone-binding protein-like (*Musa acuminata* subsp. malaccensis)	20 868.93	885	22	2.99	1.73	4.08	1.33	1.49	0.41
20	gi|695065476	Uncharacterized protein (*Musa acuminata* subsp. malaccensis)	16 951.9	316	6	2.66	0.79	3.75	1.96	1.36	0.34
21	gi|1326182	1-Aminocyclopropane-1-carboxylate oxidase (*Musa acuminata* AAA Group)	36 208.74	306	13	3.58	0.37	5.37	1.72	1.53	0.64
22	gi|695004811	Iisoflavone reductase-like protein (*Musa acuminata* subsp. malaccensis)	34 070.46	4426	96	1.97	0.46	2.44	0.48	1.24	0.05
23	gi|695047794	60S Ribosomal protein L30-like (*Musa acuminata* subsp. malaccensis)	12 506.98	209	4	2.23	1.51	2.98	1.76	1.39	0.15
24	gi|695025573	Allene oxide synthase 2-like (*Musa acuminata* subsp. malaccensis)	54 314.66	127	2	1.69	1.46	2.00	1.52	1.27	0.20
											
*Cluster 2 (16)*											
25	gi|83722842	Pectin methyl esterase (PME)/invertase inhibitor-like protein (*Musa acuminata*)	20 593.01	336	7	4.67	5.12	2.46	2.01	0.73	0.37
26	gi|695048925	Alpha-amylase 3, chloroplastic (*Musa acuminata* subsp. malaccensis)	10 7165.4	274	22	4.53	1.85	2.63	0.41	0.66	0.36
27	gi|695078082	4-Coumarate--CoA ligase-like 10 (*Musa acuminata* subsp. malaccensis)	55 761.83	177	3	5.01	3.67	4.00	2.01	0.89	0.25
28	gi|695028903	Bifunctional L-3-cyanoalanine synthase/cysteine synthase 2, mitochondrial (*Musa acuminata* subsp. malaccensis)	39 758.09	102	4	2.61	0.44	2.34	0.81	0.93	0.47
29	gi|695008336	Cationic peroxidase SPC4-like (*Musa acuminata* subsp. malaccensis)	38 263.96	67	4	2.73	0.29	2.58	1.06	0.93	0.29
30	gi|695038119	Probable carboxylesterase 15 (*Musa acuminata* subsp. malaccensis)	36 552.01	198	6	6.95	0.16	5.21	0.33	0.75	0.03
31	gi|695011158	Alpha-glucan water dikinase, chloroplastic isoform X1 (*Musa acuminata* subsp. malaccensis)	167 012.3	410	28	2.24	0.46	1.97	0.05	0.90	0.16
32	gi|695019540	Carbonyl reductase (NADPH) 2 isoform X1 (*Musa acuminata* subsp. malaccensis)	29 670.51	140	7	2.27	0.52	1.96	0.23	0.87	0.10
33	gi|695048782	Allene oxide synthase 2-like (*Musa acuminata* subsp. malaccensis)	53 716.27	112	5	2.17	0.77	1.80	0.26	0.86	0.19
34	gi|695044708	Patatin-like protein 2 (*Musa acuminata* subsp. malaccensis)	45 457.98	104	9	2.10	0.32	1.74	0.49	0.86	0.37
35	gi|12001828	1-Aminocyclopropane-1-carboxylate oxidase (*Musa acuminata* AAA Group)	34 760.01	245	17	5.48	3.40	2.14	0.76	0.54	0.47
36	gi|6606532	Pectate lyase 1 (*Musa acuminata* AAA Group)	45 277.51	440	20	2.66	1.22	1.58	0.08	0.66	0.27
37	gi|291276233	Chloroplast beta-amylase (*Musa acuminata* AAA Group)	58 123.58	165	7	4.94	4.33	1.40	0.31	0.42	0.30
38	gi|10180023	Ubiquitin/ribosomal protein 27a (Prunus avium)	17 849.02	195	8	2.38	1.66	1.29	0.13	0.69	0.43
39	gi|695041396	Prohibitin-1, mitochondrial-like isoform X1 (*Musa acuminata* subsp. malaccensis)	32 091.69	138	6	2.55	2.58	0.82	0.14	0.72	0.78
40	gi|695061190	Cysteine desulfurase 1, mitochondrial (*Musa acuminata* subsp. malaccensis)	50 174.36	124	3	2.06	0.11	0.99	0.36	0.48	0.20
											
*Cluster 3 (8)*											
41	gi|20513433	Chitinase class I (Taxodium distichum)	10 006.37	472	19	0.73	0.30	5.39	2.95	8.96	7.71
42	gi|694994696	Cinnamyl alcohol dehydrogenase 1 (*Musa acuminata* subsp. malaccensis)	39 306.07	128	5	0.84	0.15	1.91	1.31	2.44	1.99
43	gi|695061056	Cysteine synthase-like (*Musa acuminata* subsp. malaccensis)	34 047.46	235	24	0.92	0.35	2.01	1.54	2.01	0.91
44	gi|695009779	Villin-2-like (*Musa acuminata* subsp. malaccensis)	105 919.8	196	4	0.68	0.31	1.43	0.31	2.22	0.57
45	gi|695045827	Leucine--tRNA ligase, cytoplasmic (*Musa acuminata* subsp. malaccensis)	125 534.8	84	4	0.67	0.41	1.07	0.61	2.29	2.30
46	gi|189345429	CP47 protein, partial (chloroplast) (Asplenium viride)	54 629.05	422	15	0.53	0.53	0.83	0.10	2.85	2.63
47	gi|50299472	Calmodulin (Salvia miltiorrhiza)	16 777.33	449	11	0.17	0.07	0.98	0.53	5.60	0.93
48	gi|695047002	Uncharacterized protein LOC103993135 (*Musa acuminata* subsp. malaccensis)	25 047.58	252	5	0.49	0.14	0.84	0.58	1.97	1.76
											
*Cluster 4 (7)*											
49	gi|695060249	Probable sucrose-phosphate synthase 1 isoform X1 (*Musa acuminata* subsp. malaccensis)	119 201.2	76	4	1.35	0.45	0.65	0.36	0.46	0.11
50	gi|695054381	Probable xyloglucan endotransglucosylase/hydrolase protein 7 (*Musa acuminata* subsp. malaccensis)	32 871.67	358	14	1.55	1.10	0.48	0.05	0.43	0.33
51	gi|695036639	Serine hydroxymethyltransferase, mitochondrial-like (*Musa acuminata* subsp. malaccensis)	57 592.95	185	6	1.22	0.50	0.47	0.19	0.46	0.34
52	gi|376341422	Small heat shock protein (*Musa acuminata* AAA Group)	17 746.54	349	12	1.32	0.50	0.43	0.01	0.35	0.14
53	gi|695075128	Proliferation-associated protein 2G4-like (*Musa acuminata* subsp. malaccensis)	43 804.25	145	7	1.06	0.22	0.43	0.09	0.43	0.17
54	gi|410634	Cytochrome c reductase-processing peptidase subunit II, MPP subunit II, P53 (potatoes, var. Marfona, tuber, Peptide Mitochondrial, 530 aa)	59 463.69	136	4	1.12	1.01	0.28	0.21	0.28	0.06
55	gi|695067061	17.3 kDa class II heat shock protein-like (*Musa acuminata* subsp. malaccensis)	17 626.59	795	23	1.19	0.34	0.35	0.00	0.31	0.09
											
*Cluster 5 (12)*											
56	gi|695006926	Heme-binding protein 2 (*Musa acuminata* subsp. malaccensis)	24 202.41	223	9	0.77	0.33	0.44	0.07	0.60	0.16
57	gi|695029002	Glycerate dehydrogenase isoform X1 (*Musa acuminata* subsp. malaccensis)	42 297.46	251	10	0.81	0.19	0.44	0.21	0.59	0.40
58	gi|695051607	Protein DJ-1 homolog A (*Musa acuminata* subsp. malaccensis)	47 213.36	142	3	0.76	0.16	0.38	0.02	0.52	0.14
59	gi|695055384	GEM-like protein 5 (*Musa acuminata* subsp. malaccensis)	28 200.4	73	3	0.80	0.24	0.38	0.38	0.57	0.65
60	gi|695046031	Ketol-acid reductoisomerase, chloroplastic-like (*Musa acuminata* subsp. malaccensis)	63 901	856	39	0.90	0.15	0.50	0.11	0.57	0.22
61	gi|695018933	Glycine-rich RNA-binding protein 7-like (*Musa acuminata* subsp. malaccensis)	16 140.95	225	11	0.89	0.03	0.35	0.22	0.40	0.27
62	gi|657384878	Peroxidase family protein (Medicago truncatula)	38 096.33	84	4	0.68	0.42	0.34	0.28	0.78	0.90
63	gi|695010609	Peptidyl-prolyl cis-trans isomerase-like (*Musa acuminata* subsp. malaccensis)	18 575.62	1718	58	0.62	0.05	0.43	0.15	0.68	0.19
64	gi|149391329	Mitochondrial formate dehydrogenase 1 precursor (Oryza sativa Indica Group)	15 437.19	93	5	0.76	0.20	0.46	0.38	0.70	0.69
65	gi|695060599	Triosephosphate isomerase, chloroplastic-like (*Musa acuminata* subsp. malaccensis)	33 611.81	421	19	0.70	0.15	0.49	0.23	0.68	0.18
66	gi|695067452	Outer envelope pore protein 16, chloroplastic (*Musa acuminata* subsp. malaccensis)	15 497.52	209	8	0.66	0.03	0.41	0.10	0.63	0.18
67	gi|197691939	33 kDa precursor protein of the oxygen-evolving complex (Salicornia europaea)	35 196.39	165	9	0.58	0.03	0.29	0.21	0.51	0.38
											
*Cluster 6 (6)*											
68	gi|695022172	Oxygen-evolving enhancer protein 3-1, chloroplastic-like (*Musa acuminata* subsp. malaccensis)	24 720.82	212	14	0.49	0.04	0.65	0.21	1.32	0.33
69	gi|399162600	Glutathione peroxidase (*Musa acuminata* AAA Group)	18 691.94	374	13	0.37	0.16	0.47	0.21	1.27	0.01
70	gi|225466690	Sedoheptulose-1,7-bisphosphatase, chloroplastic (Vitis vinifera)	42 898.17	98	3	0.43	0.26	0.44	0.17	1.12	0.27
71	gi|672114779	Catalase isozyme 2-like (*Phoenix dactylifera*)	56 378.56	1627	36	0.40	0.17	0.41	0.01	1.10	0.43
72	gi|695038951	Probable linoleate 9S-lipoxygenase 4 (*Musa acuminata* subsp. malaccensis)	76 399	253	17	0.40	0.16	0.43	0.02	1.20	0.55
73	gi|694999079	Probable linoleate 9S-lipoxygenase 5 (*Musa acuminata* subsp. malaccensis)	98 781.88	1498	85	0.18	0.03	0.24	0.08	1.29	0.21
											
*Cluster 7 (18)*											
74	gi|76574236	Chloroplast ribulose-1,5-bisphosphate carboxylase/oxygenase small subunit (*Musa acuminata* AAA Group)	20 807.84	208	10	0.45	0.29	0.43	0.24	1.00	0.11
75	gi|339736137	Ribulose-1,5-bisphosphate carboxylase/oxygenase large subunit (Ostreobium quekettii)	15 095.4	221	10	0.52	0.21	0.48	0.12	0.96	0.17
76	gi|146724107	Chloroplast ribulose-1,5-bisphosphate carboxylase/oxygenase small subunit (*Musa acuminata* AAA Group)	20 849.92	566	28	0.48	0.24	0.44	0.13	0.97	0.22
77	gi|695080392	Linoleate 9S-lipoxygenase A-like (*Musa acuminata* subsp. malaccensis)	96 773.43	85	6	0.43	0.12	0.38	0.07	0.90	0.09
78	gi|695040260	Oxygen-evolving enhancer protein 2, chloroplastic-like (*Musa acuminata* subsp. malaccensis)	28 178.71	1284	26	0.32	0.20	0.26	0.12	0.87	0.15
79	gi|695051060	Fructose-bisphosphate aldolase, chloroplastic-like (*Musa acuminata* subsp. malaccensis)	42 279.08	313	14	0.58	0.29	0.48	0.12	0.88	0.24
80	gi|695058993	Cysteine proteinase 2-like (*Musa acuminata* subsp. malaccensis)	34 996.01	389	18	0.54	0.17	0.49	0.29	0.86	0.28
81	gi|409900388	Copper/zinc superoxide dismutase CSD2B-1 (*Musa acuminata*)	22 048.99	107	6	0.53	0.09	0.46	0.15	0.85	0.13
82	gi|1076373	Photosystem II oxygen-evolving complex protein 2—Arabidopsis thaliana (fragment)	1434.2	163	8	0.23	0.06	0.17	0.11	0.69	0.30
83	gi|695039702	40S Ribosomal protein S19 (*Musa acuminata* subsp. malaccensis)	16 103.94	273	16	0.62	0.21	0.49	0.30	0.75	0.24
84	gi|1770115	Ribulose-1,5-bisphosphate carboxylase/oxygenase large subunit (Casasia clusiifolia)	52 706.64	1337	51	0.57	0.28	0.38	0.08	0.73	0.22
85	gi|22859628	Ribulose-1,5-bisphosphate carboxylase/oxygenase, partial (chloroplast) (*Selaginella pilifera*)	47 457.51	1470	58	0.53	0.13	0.36	0.04	0.71	0.25
86	gi|28076780	Ribulose-1,5-bisphosphate carboxylase/oxygenase large subunit (*Ulva lactuca*)	50 182.23	1185	40	0.53	0.21	0.36	0.03	0.74	0.24
87	gi|695039506	Phospholipase D alpha 1-like (*Musa acuminata* subsp. malaccensis)	93 215.93	98	3	0.59	0.13	0.48	0.28	0.77	0.31
88	gi|672128345	Oxygen-evolving enhancer protein 1, chloroplastic-like (*Phoenix dactylifera*)	35 656.52	522	12	0.50	0.16	0.39	0.35	0.71	0.47
89	gi|548603	Photosystem I reaction center subunit II (Hordeum vulgare)	21 976.88	501	20	0.54	0.07	0.43	0.02	0.79	0.14
90	gi|695048996	Oxygen-evolving enhancer protein 1, chloroplastic-like (*Musa acuminata* subsp. malaccensis)	36 229.49	350	15	0.34	0.16	0.23	0.17	0.63	0.20
91	gi|672162172	Oxygen-evolving enhancer protein 1, chloroplastic (*Phoenix dactylifera*)	35 547.58	465	11	0.33	0.10	0.22	0.17	0.62	0.31
